# Crowdsourcing food security: introducing food choice derivatives for sustainability

**DOI:** 10.1007/s12571-023-01363-7

**Published:** 2023-04-26

**Authors:** Hana Trollman, Sandeep Jagtap, Frank Trollman

**Affiliations:** 1 Department of Work, Employment, Management and Organisations, School of Business, University of Leicester, University Road, Leicester, LE1 7RH UK; 2 Sustainable Manufacturing Systems Centre, School of Aerospace, Transport & Manufacturing, Cranfield University, Cranfield, MK43 0AL UK; 3 Glenfield Hospital, University Hospitals of Leicester NHS Trust, Leicester, LE3 9QP UK

**Keywords:** Crowdsource, Derivatives market, Food security, Food waste, Sustainability

## Abstract

Global food supply chains are unprepared for the increasing number and severity of the expected environmental, social and economic shocks in the coming years. The price-setting process of commodities is directly impacted by such shocks, influencing consumer behavior regarding food choice and consumption. Both the market and advances in precision agriculture drive increased production and consumption. However, there has been a lack of consideration of how consumer behavior could be harnessed to mitigate such shocks through decreased consumption and reduced waste. The SAPPhIRE model of causality was applied to design sustainable and ecologically embedded futures derivatives that could have a role in affecting commodity markets. Multi-agent systems were combined with artificial intelligence and edge computing to provide the necessary functionality. The impact of war in Ukraine was used to exemplify the design of consumer “food choice” derivatives. This resulted in a mechanism to bring aggregated acts of consumer compassion and sustainability to commodities markets to mitigate food security shocks. When implementing food choice derivatives, care must be taken to ensure that consumer food choices are rational and compatible with individual nutritional needs and financial situations, and that the legitimate interests of agri-food businesses are protected.

## Introduction

Global food crises and their associated food price increases worsen food insecurity and drive people to reduce their food consumption (Gustafson, 2013; Suppan, 2008). Undernourished people are present in both developed and developing countries, making Sustainable Development Goal 2, which aims to achieve “zero hunger”, globally relevant and a key issue for policymakers. Factors such as increasing food demand in some countries, subsidies for biofuels, rising costs of cultivation, crop failure, reduced grain stockpiles and inadequate policy support for agriculture do not completely explain short-term food price rises (FAO, 2017).

The relationship between food supply and demand is distorted by financial derivatives markets (Marquina, 2013; Xuan et al., 2022), affecting the food security of nations. Disagreements exist among economists and policymakers about the role of commodity futures (food speculations) in the price hikes of 2007/mid-2008 and 2010/2011. Empirical results indicate that commodity futures have a negative impact on the food security of low-income countries, and financial crises exacerbate the negative effects of some commodity futures on food security for low-, middle- and high-income countries (Sosoo et al., 2021).

The causal relationship between commodity futures and commodity prices remains under debate. The expectation has been that commodity speculation would be stabilizing, reducing the extent of commodity price variations, and that this would help farmers (producers) and processors hedge against short-term unfavorable price movements (Suppan, 2008). The market was expected to always be in backwardation (with the prices of futures contracts always lower than the prices of spot contracts). However, the phenomenon of price rise fluctuations of agricultural products is not adequately explained by traditional supply and demand theory (Aït-Youcef, 2019; Algieri, 2016).

Cereal-based supply chains may be described by a generic Input-Process-Output (IPO) model with five links: farms, storage areas, mills, food processing plants and consumers as the final actors (Carvalho et al., 2021). Advances in precision agriculture increase the yield and profitability of crops while reducing the resources necessary for cultivation (Van Evert et al., 2017; Zhang et al., 2002). Ollenburger et al. (2022) describe the challenges of realistically estimating crop yields for agro-economic modelling as they depend on both biophysical characteristics (e.g., soil, climate, etc.) and local crop management practices (e.g., mechanization, irrigation, crop cultivars). Consequently, even empirically based food speculation is unlikely to have a high degree of accuracy, becoming even less accurate in the presence of unforeseen food shocks.

Assumptions about economic growth affect commodity market outlooks. For example, if China remains a strong grain importer, there could be a 4 to 25% increase in agricultural commodity prices compared to projections in the OECD-FAO Agricultural Outlook 2021–2030 (Adenauer, 2022; Kavallari et al. 2014). Public buffer stocks are held by many countries for price stabilization and food security, but these are rarely effective (Beaujeu, 2016). To counter food insecurity, the establishment of an independent global buffer stock to create a food reserve has been proposed, but there are numerous obstacles to practical implementation, especially in the identification of appropriate price triggers (Gilbert, 2011; Tangermann, 2011; Wright, 2009). Other proposed solutions include the use of futures contracts or options (as well as forward contracting) as tools to manage price risks (e.g., Gilbert (2011), Sarris (2010), Sarris et al. (2010); Tangermann (2011)) or an International Grain Clearing Arrangement with the objective of guaranteeing grain import contracts between private and public agents (Sarris et al., 2010; Tangermann, 2011).

Strictly financial interventions in commodity futures markets may lack sustainability considerations. Integrating environmental, economic, and social attributes has increased in popularity when selecting suppliers and sourcing processes (Azadnia et al., 2015; Ghadimi et al., 2018, 2019) which become part of price setting. A multi-agent system (MAS) has been proposed to support supplier selection based on supplier sustainability informed by a deep Q-learning agent for agricultural future market price forecasting (Pérez-Pons et al., 2021). Mechanisms for crowdsourcing societal tradeoffs as part of computational social choice have been proposed (Conitzer et al., 2015). A holistic consideration of solutions in terms of ecological embeddedness (benefits for economic actors and the environment) across the value chain would be desirable (Trollman & Colwill, 2020).

This research employs a modified version of the SAPPhIRE model of causality (Chakrabarti et al., 2005) which includes virtual as well as physical considerations to design a food choice derivative that could counter negative commodity market fluctuations during periods of crisis. Constraints on the design include ecological embeddedness and sustainability considerations. The application of the design is exemplified on the case of Ukrainian wheat in regards to the invasion of Russian forces in February 2022. Proposed approaches to implementation are suggested based on crowdsourcing platforms.

Few studies investigate economic motivations in consumer food choice (Martinho et al., 2022). Due to increased supply-side volatility as well as reduced willingness of wealthier households to reduce consumption in the face of shortages, commodity markets are likely to become more volatile in the future (Baldos & Hertel, 2015). Consumers are normally considered to be price-takers with economic motivations significantly affecting their behavior regarding food choice and subsequent food consumption (Martinho et al., 2022). In fact, nearly every market participant, producer or consumer, is a price-taker. The price-maker is the speculative positioning in the regulated futures markets - the collective actions of technical traders who buy on rising prices and sell on declining prices. Consequently, the question this research poses is: How can an alternative consumer-based price-making mechanism be introduced to the derivatives market to mitigate food speculation, improving food security and the sustainability of agricultural production?

The sections below initially present the relevant background gathered to support the design phase (literature review), a description of the methodology, the results of the application of the design phase, and the exemplification of the selected case.

## Literature review - Supporting information for the design phase

Agri-food systems are embedded in complex ecological, economic, and social processes through dynamic interactions that are vulnerable to short-term shocks and long-term stresses (Thompson & Scoones, 2009). Decreasing food waste would enable the more sustainable feeding of the world’s population. Research supports place-based solutions that are locally relevant to reducing household food waste in advancing sustainable food systems (Ahmed et al., 2021).

### Cereals value chain

**Fig. 1:**
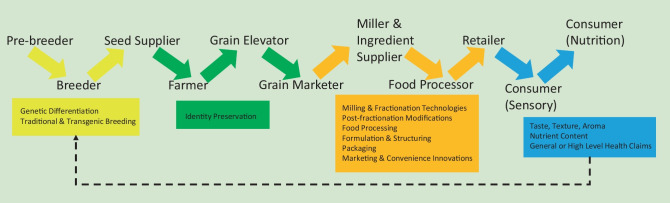
Schematic representation of a “typical” cereal value chain. (adapted from Morell (2012))

A typical cereal value chain is shown in Fig. 1. However, this value chain does not capture agricultural wastes, co-products and by-products (AWCB) that are produced (Ćosić et al., 2016) or the amount of food wasted (Jeswani et al., 2021). Post-consumer food waste contributes more in terms of both quantity and environmental impact than other life cycle stages (primary production, food processing and distribution), however, these also have a significant impact. To release cereals for redistribution and minimize wastes, the grains need to be at the primary production stage (harvest and storage). Cereals that are milled and converted to ingredients have a shorter shelf life and added value that is not part of commodity markets. For ecological embeddedness, information about consumer food choice consequently needs to be communicated upstream to ensure that all the actors in the value chain can benefit.

### Derivatives in agricultural production

The derivatives market is for financial instruments such as contracts for futures, options, forwards and swaps whose value is derived from their underlying assets. Futures contracts involve both a right and obligation to carry out the contract as agreed, and are standardized, which means they are traded on exchange markets unlike other derivatives. Futures prices are the price of a commodity traded on a futures exchange representing a contract to pay a predetermined price at a set delivery date in the future. The price information for futures is collected from a range of global marketplaces. The extreme volatility in the price level of wheat results from a mix of factors including speculation, global demand, and real effective exchange rates with speculation being an important determinant of price dynamics (Algieri, 2016). Speculative activities often exceed the level required to offset unbalanced hedging, resulting in market destabilization (Algieri, 2016).

Farmers risk losing money if the price of their crop falls before harvest and sale. Futures markets are an important source of price information for farmers although only a small percentage of farmers directly trade futures (Pérez-Pons et al., 2021). Farmers may minimize risk by selling futures contracts which guarantee the receipt of the predetermined price. In place of delivery, futures contracts may be liquidated by offsetting (selling the commodity locally on the spot market). Physical delivery is relatively rare as the contracts of buyers and sellers are counterbalanced. Hedging is beneficial to the economy because actors in the value chain such as farmers and millers have greater certainty about how much they will earn and pay for commodities.

The participants in derivatives markets are hedgers, speculators, arbitrageurs, and margin traders. Speculators have no interest in actually selling or purchasing the physical commodity. Derivatives trading is complex so the general public mostly avoid participation. Farmers may use a grain merchant or co-op to access futures, or go directly to a futures broker. Relatively few brokers will manage individual accounts like those of farmers, and margin payments to cover exposure when the market moves unfavorably are required along with possible top-ups. For many small and marginal farmers, Farmer Producer Organizations (FPOs) can act to procure and aggregate commodities, ensuing that the size and quality standards required for agri-futures trade are met. India has set a target of creating 10,000 FPOs by 2024 (Chatterjee et al., 2019). Similarly, consumer participation in derivatives markets would need to be enabled through aggregation.

### Food insecurity and consumer behavior

With shocks such as extreme weather events predicted to significantly damage crops, corresponding increased commodity price volatility is anticipated (Baldos & Hertel, 2015; Suppan, 2022). A strong incentive exists to shield urban consumers from world price spikes (Anderson et al., 2013) to avoid urban unrest. Food insecurity and dietary behaviors, including food choices and preparation methods, are infrequently examined in the literature. Food insecurity has been associated with lower nutrient intake and higher fat intake with lower frequency of fat-lowering behaviors (Mello et al., 2011). When food prices rise, the real incomes of urban, wage labor dependent households fall sharply, necessitating a cut to food consumption (Ahmed et al., 2009). For wealthier households, there may be diminished willingness to curb food consumption (Baldos & Hertel, 2015). The demand-induced scarcity of wheat flour and eggs during the Covid-19 pandemic in the United Kingdom (UK) indicated that consumers are creatures of habit, unlikely to make sustainable and nutritious substitutions or alternative food choices (Trollman et al., 2021) and options such as entomophagy remain stigmatized (Adegboye et al., 2021).

Concurrently, effective evidence-based consumer food waste reduction strategies have been implemented in European countries (Parry et al., 2014; Schmidt, 2016). There is also evidence that consumers are willing to take additional action to reduce food waste (Ahmed et al., 2021). However, the relationship between voluntarily reduced consumption accompanied by temporary dietary change in support of food waste reduction for humanitarian purposes lacks investigation as there is no direct enabling mechanism. The design of such a mechanism is presented in the following sections.

## Methods

The overarching method used in this research is that of innovative abduction in design (Roozenburg, 1993), extended by Kroll & Koskela (2015, 2017). Abductive reasoning examines facts to suggest a theory by generating innovative ideas depending on recursive logic based on knowledge and the ability to analogically associate different domains (Calabrese & Costa, 2015). In engineering design, generating principal solutions is considered to be a crucial phase in the design process (the kernel of design). At the most basic level, the solution to a design problem comprises of a description of form and its actuation for a given purpose or outcome (Roozenburg, 1993). The purpose identified for this research was to design a financial instrument to counteract short-term market fluctuations caused by shocks to food security.

Abduction-based futures research moves from closed, imaginary future states to alternative, open explanations (Patokorpi & Ahvenainen, 2009). Design research relies on basic research to develop technical norms which can be used to improve human activities. Futures studies are an instrument for design which tend towards systemic analysis when complexity is encountered. The solution to such problems may be seen as the identification of an opportunity, but opportunities typically cannot be recognized from inside or outside an existing framework. Futures research based on the abductive method breaks free of summary and collective knowledge which enables completely new futures designed not only from an existing state, but also via anomalies, imaginary explanations, and new theoretical frameworks (Patokorpi & Ahvenainen, 2009). The identification of novel or surprising findings is a key component of abduction which can then be used to extend, advance, or revise existing theories (Halpin & Richard, 2021).

According to Tavory and Timmermans (2014), an abductive study should answer three questions: (1) Does the data fit the theory and/or conclusions? (2) Are the findings plausible, or is there another more plausible explanation? (3) If the findings are accurate, why do they matter? Answering the first question requires transparency in the collected information/data; the second question involves constantly interrogating the analysis, seeking alternative explanations, and relating these alternatives to the data; and finally, the third question is answered by connecting findings to previous studies and theory, and by demonstrating scope or generalizability.

Abductive reasoning recognizes the role of the researcher as an unavoidable and essential element in the analytical dynamic between the researcher and the subject under study (Thomas, 2010) as part of a methodological approach that does not undermine, but enhances the research, particularly for case studies (Conaty, 2021). Case study research supports depth of interaction between the researcher and collected information, hence resonating with abduction as a methodological approach. In considering the “how” research question as part of the abductive process, the case study may be considered both descriptive and explanatory (Conaty, 2021). Studies based on a single case study, while facilitating phronesis and depth of understanding, are open to criticism for lack of generalizability; however, it has been argued that any weakness of case study due to generalizability fails to recognize both the limits of induction and to acknowledge the significance of abduction (Thomas, 2010). The researcher plays the important role of being an instrument of the method (Wa-Mbaleka, 2020) and the primary instrument of sensemaking for the study (Barrett, 2007).

Function–behavior–structure models such as the SAPPhIRE model are intended to capture rich causal descriptions suitable for the purposes of researchers. The SAPPhIRE model is a generic model for representing causality of natural and artificial systems to help develop novel ideas to solve design problems, as illustrated by using databases of natural systems and artificial mechanical systems to describe various behaviors (Chakrabarti et al., 2005). The same behavior can be achieved and realized by different solution forms (Hubka & Eder, 2012) which rely on information from the databases. In this research, no databases exist for selecting mechanisms. Consequently, innovative abduction is employed.

**Fig. 2:**
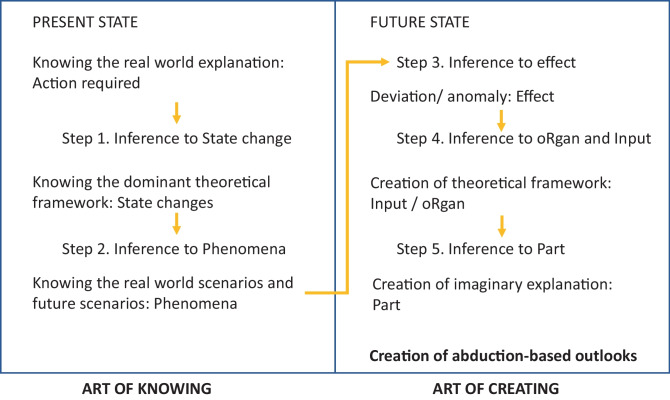
Five-step model of abduction incorporating the process framework of the abductive approach (Patokorpi & Ahvenainen, 2009)

A modified version of the SAPPhIRE model of causality was applied to understand the behavior of financial derivatives and their role in affecting commodity markets. SAPPhIRE is a model of causality consisting of seven elementary constructs: States, Actions, Parts, Phenomena, Inputs, oRgans and Effects (Chakrabarti et al., 2005). The SAPPhIRE constructs were applied as part of a Five-step model of abduction (which in some cases may involve fewer steps when more constructs are combined) (Bhatt et al., 2021) as illustrated in Fig. 2.

As noted by Bhatt et al. (2021), after the generation of the various constructs, the alternatives may be evaluated against given criteria (requirements) such as economic criteria. The requirements imposed in this research were sustainability and ecological embeddedness of the value chain, as described in the Introduction. Therefore, before the SaPPhIRE model was applied, the value chain for cereals was first described along with sustainability implications and routes to ecological embeddedness (Trollman et al., 2020) as found in the Literature Review. This corresponds to a first phase of Radical Innovation Design (RID), intended to provide an in-depth understanding of the current situation which is then followed by the design phase that generates new objects to improve the situation (Lamé et al., 2018). Finally, following the development of food choice derivatives in the design phase, a case example of the effect of the war in Ukraine on wheat intended for North Africa is presented to illustrate how the crowdsourcing of food security could be realized. The case approach method adopted follows Zucchella & Urban (2014) in that the abductive approach then tests the hypothesis on a case study.

## Results

### Application of the design phase – the SAPPhIRE model

The application of SAPPhIRE in this research is constrained by sustainability and ecological embeddedness considerations for the value chain. For reasons of sustainability, increasing production to counter shocks to food security is infeasible over both the short-term (due to the time between sowing and harvest) and the longer-term (due to resource limits, biodiversity concerns and societal impacts). Consequently, efficiencies need to be found within the system e.g., food waste. For ecological embeddedness of the value chain, technology may be employed to facilitate the necessary information transfers to create benefits to both the economic actors and the environment. Examples of these benefits are described in the case study which follows.

- Step 1: Inference to State change
  The State is comprised of the attributes and values of attributes that define the properties of the system at a given moment of time. Input for one system may be a state change for another such that the viewpoints are created by system boundaries (Chakrabarti et al., 2005). The State in question corresponds to that of the futures commodity market (a dynamic state) as influenced by various attributes including speculation, global demand, and real effective exchange rates.
- Step 2: Inference to Phenomenon
  The Phenomenon is a set of potential changes associated with a physical effect for a given oRgan and Inputs. The Phenomenon is the countering of a shock to the system as may be caused by climate-related crop failure, labor shortages due to a pandemic and/or war in a globally important food producing nation.
- Step 3: Inference to Effect
  The Effect reflects laws governing change. These may be laws of nature or constraints imposed by regulations. The Effect herein is moderation of commodity market fluctuations as governed by transactions on the commodity markets.
- Step 4: Inference to oRgan and Input
  An oRgan is a structural context necessary for an effect to be activated. Input is the information or material requirements to activate the Effect. The oRgan is the proposed food choice derivative. The Input is the information about the participation of consumers in creating the oRgan.
- Step 5: Inference to Part
  The Part is the set of physical components and interfaces constituting the system and its environment of interaction. Parts create or enable oRgans. In this case, Parts are the enablers of consumer food choices, e.g., the crowdsourcing platform aggregating decreased consumption and food waste reductions.

### Case study: War in Ukraine

Russia’s invasion of Ukraine is anticipated to cause a “perfect storm” of increased food prices leading to civil war across the developing world (Jagtap et al., 2022; Koren & Winecoff, 2022). Ukraine produces about 6% of all food calories traded on the international market (Amis Market Monitor, 2022). Due to the invasion by Russian forces in February 2022, Ukraine is expected to harvest less than half of the 80 MMT (million metric tonnes) of grain (wheat, corn and barley) produced in 2021. The affected July harvest of wheat in Ukraine results from planting in March and February with exports of about 16.7 MMT intended primarily for North Africa and South Asia (OEC, n.d.). Indonesia, Egypt, Pakistan, Bangladesh and Morocco (Reidy 2022) each expected over 1 MMT of Ukrainian wheat in 2022.

Bread production globally is estimated at about 100 MMT per year of which 65% is consumed in Europe (Melikoglu & Webb, 2013). About 25% of all bread in the Netherlands is wasted (about 800 thousand loaves every day) (Rietveld, 2019). In the UK, about half of the bread produced is wasted. The amount of bread wasted every day is about 1 million loaves (WRAP, 2021) or about 500 MT of wheat per day (based on a loaf with 400 g of wheat flour made from 500 g of wheat). Therefore, if no bread was wasted in July 2022 (31 days) in the UK, about 15,500 MT of wheat could be added to global commodity markets. This is sufficient to trade on wheat futures exchanges (Chicago Board of Trade (CBOT) and NYSE Euronext (Euronext)) as shown in Table 1.

**Table 1:** Contract sizes for wheat futures exchanges

Exchange and Product Name	Symbol	Contract Size
CBOT Wheat Futures	W	5000 bushels (136 MT)
Euronext Milling Wheat Futures	EBM	50 MT
Euronext Wheat (No. 405) Futures	WHT	100 MT

**Fig. 3:**
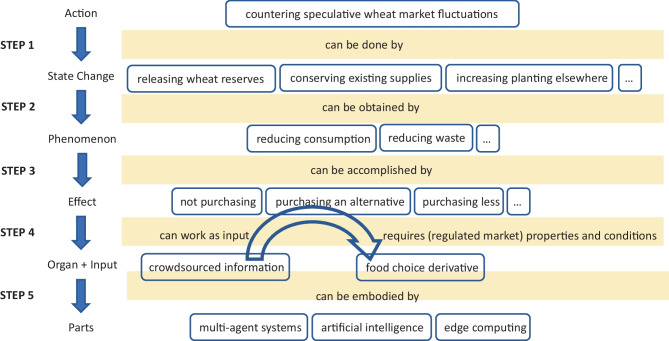
Wheat market case study reasoning with SAPPhIRE model

Application of the SAPPhIRE model to wheat markets is shown in Fig. 3. In Step 1, there are numerous strategies that may be employed to counter speculative wheat market fluctuations, not all of which are noted in Fig. 3 as indicated by “…”. The conservation of existing supplies is selected due to the constraint of sustainability as previously explained. The conservation of existing supplies of wheat can thus be accomplished by reducing consumption or reducing waste as enabled by consumers not purchasing wheat-based products such as bread within a specified limited time, purchasing an alternative product that is not wheat-based during that time, and/or purchasing less of the wheat-based product. The information about consumer food choices then needs to be conveyed upstream the supply chain for ecological embeddedness: so that the supply chain actors can prepare and benefit, and so that the grain is physically available in pre-processed form. The food choice derivative can consequently be created based on the amounts of wheat conserved.

Examples of how consumers could purchase less include schemes such as:

- Buy one, get one later (after the specified time) for those who purchase more than one loaf regularly.
- Buy half a loaf (enabled by retailer and/or packaging of manufacturer).
- Buy none, get one later (the negative coupon: a guarantee for the future in case of local shortages).
- Buy none, get a nutritionally equivalent alternative.
- Buy none, get a functionally equivalent alternative.

Such schemes would support ecologically embedded benefits to the retailer in terms of customer loyalty and stock optimization, to the consumer in terms of health benefits which could also inform the seed prebreeder and breeder, and to the rest of the value chain with respect to the ability to take proactive action.

An MAS is a computerized system composed of multiple interacting intelligent agents. Each agent has autonomy and flexibility, making multi-agent technology suitable for distributed, real-time applications. MASs may be used for cognitive modeling and social simulation (Sun & Naveh, 2004). MAS has been combined with AI and blockchain for privacy and security of health records (Alruwaili, 2020). The privacy and security of consumer food choices could thus be protected and supported by edge computing which offers an efficient alternative to storing, processing, and analyzing consumer data. Crowdsourcing enables consumer engagement and therefore improves the likelihood of success: to decide which type of purchasing scheme should be offered, to connect people to buy the other half of the loaf of bread, to make decision about the timing and location of purchases, select nutritional and functional alternatives, etc. which would prepare the necessary aggregated information to ensure the standards for futures trading are met. The ultimate goal would be global benefits throughout the value chain with lower and more stable food and raw ingredient prices.

**Fig. 4:**
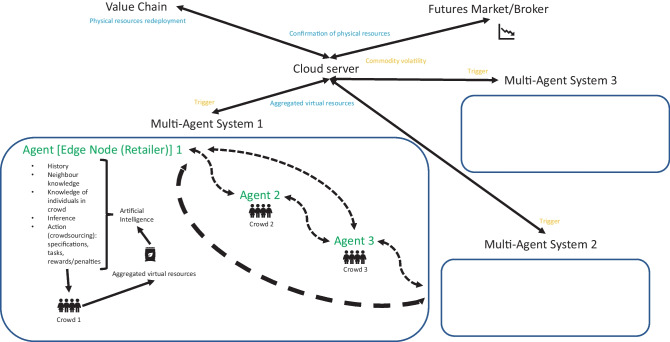
Possible crowdsourcing system illustrating interaction of MASs and aggregated virtual resources with physical resources and futures markets. System informed by Jiang et al. (2018) and Hosseini et al. (2019)

Crowdsourcing is usually defined as the practice of collecting and aggregating needed services, information or other resources supplied by the general public (Hosseini et al., 2015). Crowdsourcing applications have the following key elements: (1) tasks, which are outsourced with various characteristics in reality e.g., the purchasing schemes above; (2) requesters, who release the tasks; (3) system platforms, which provide efficient measures to manage and organize the entire crowdsourcing process; and (4) workers, i.e., the crowd of people (Jiang et al., 2018). The power in food supply chains lies with retailers who are in the position of managing various purchase schemes for the benefit of their stock. Retailers would be encouraged to participate also for reasons of good corporate responsibility. Retailers are consequently a logical choice for representing requesters who at the same time would be agents in the MAS, capable of making autonomous decisions (Dorri et al., 2018) supported by AI analysis of consumer behavior, yet acting collaboratively with other agents to aggregate information on reduced consumption to support the food choice derivatives. A typical feature of MAS is that the real benefit of agents can only be harnessed through collaborative work with other agents through features such as sociability (sharing knowledge), autonomy (independent execution of decision-making for appropriate action), and proactivity (using history, sensed parameters, and the information from other agents to predict possible future actions) (Dorri et al., 2018). MAS can be leaderless or leader-follower with mobile leaders or multiple leaders also a possibility. Each agent may work within its own edge computing environment and send collected data to other agents when pre-defined targets are met with respect to the social network (crowd) it interacts with. Information may then be communicated to other actors in the value chain to inform and enable planning in aggregate. The individual contributions would be captured from interactions of the crowd with the crowdsourcing platform via various devices (e.g., smartphones, laptops) enabling a flexible level of co-creation with the aggregating mechanism either centrally operated by the task issuer or intrinsic to a crowdsourced system (Hosseini et al., 2019). Figure 4 illustrates a possible configuration using retailers as agents who would employ specifications for stock optimization and rewards/penalties supporting consumer health and nutrition based on localized knowledge and behavior.

## Discussion

The globalization of food markets, combined with the production advances of the Green Revolution, have created a world where severe regional famine is exceptional rather than normal. As it is demonstrably true that total food production remains sufficient to meet current global nutritional needs (Rahimifard et al., 2018), institutional improvements in markets are key to providing food security to the nearly 9% of humans who are currently undernourished. While markets reward innovation, they are often slow to adopt institutional change. The London Metal Exchange banned day drinking by traders in 2019, some 142 years after sober trading was proposed.

World cereal production has been over two billion MT every year since 2004, and over one billion MT every year since 1968. Price fluctuations create winners and losers, but very large and rapid price changes can be devastating to the economy and food security. February of 2022 saw the price of wheat rise by over a hundred dollars per MT (43%) over a period of just two weeks. If all payments for cereals during the year were similarly changed, the result could be the unexpected transfer of over three hundred billion US dollars, more than the entire GDP of Chile or Finland. Sufficient liquid assets to create that kind of wealth transfer from consumers to producers do not exist, and price fluctuations on that scale could be ruinous, leaving unsold cereals and empty stomachs. A price change in the opposite direction could leave producers financially nonviable, risking the mass abandonment of agriculture. Long term contracts mitigate those risks by ensuring that significant amounts of grain have prices set at levels that leave producers viable and consumers solvent even if the current price rises or falls to beyond those limits. Derivatives markets have the potential to further stabilize the actual transfer of cereals to hungry people in the face of economic shocks.

Waste reduction is an inherently limited source because the waste is a finite subset of overall production. 100% efficiency is technically impossible and efforts to move closer to perfection have diminishing returns. There is currently every reason to believe that food waste is high enough that waste-prevention efforts are worth pursuing, but there is a limit to how large this segment of the market can grow. Waste reduction derivatives logically are restricted to being a smaller economic consideration than the underlying primary markets. This stands in contrast to value adding food preparation, which at times is valued many times the worth of the inputs.

The proposed food choice derivatives investigated are feasible both technologically and in terms of aggregating sufficient quantities of commodities for trading on futures markets in the case of wheat. However, there are many unknowns related to the actual response of consumers and futures markets. Although the food choice derivatives have been designed as a voluntary measure to address crises, given severe shocks to the food systems of nations, governmental bodies could compel the use of such a system for food redistribution to minimize malnutrition and starvation.

The proposed food choice derivatives compare favorably with other proposed solutions in that ecological embeddedness of the value chain and sustainability of the food system are intrinsic. Futures contracts and options have been proposed as tools to manage price risks (e.g., Gilbert (2011), Sarris (2010), Sarris et al. (2010), Tangermann (2011)), but these are solely financial in nature. Reducing food waste and consumption are a superior solution to increasing agricultural production due to the likelihood of insufficient time to harvest and increased social and environmental pressures connected to unsustainability. Previous research has concluded that pathways to reduce environmental impacts of the global food system should be designed around regional differences in food consumption (Ibarrola-Rivas & Nonhebel, 2022).

The case study for Ukrainian wheat indicates that the aggregated quantities from one nation such as the UK over a short period of time (one month to maintain consumer engagement) would be insufficient to replace all of the wheat harvest anticipated to be lost due to the war in Ukraine (thousands of MT as opposed to MMT), but similar efforts across the European Union would be more significant. Achievement of near full replacement of the lost harvest would be an added benefit as the defined role of the proposed food choice derivatives is not to replace the lost harvest, but to counter speculative market fluctuations that the lost harvest causes.

Consumers in times of crises may not make appropriate food choices. There are few studies that investigate the economic motivations in consumer food choice (Martinho et al., 2022). The proposed food choice derivative should introduce the option to participate in a humanitarian effort prior to any actual changes in supply or cost although consumers may perceive a future resource scarcity. Ideally, the triggers for initiating crowdsourcing based on market volatility would be set early enough to counter significant price spikes, limiting the subsequent effects on purchasing power. Financial insecurity would likely have a negative effect on charitable giving but could be positive for the proposed food choice derivatives in that consumers would already be considering reduced consumption and waste.

Previous research has shown that consumers facing limited supply may not make the best choices to benefit their health and nutrition (Maxwell et al., 2008; Trollman et al., 2021). Consumers tend to select similar substitutes with dissimilar alternatives reducing the desire for the original product (Arens & Hamilton, 2016, 2018). If consumers are asked to select alternatives or substitutes, there may be long-term effects on consumer habits, behavior and brand loyalty with the exact effects being unknown.

The use of strategies to encourage consumers to consume less bread may have positive impacts on health. There is evidence that the consumption of pulses is an important strategy to reduce the risk of cardiovascular disease (Lukus et al., 2020) – pulses (beans, peas, chickpeas, and lentils) can be used as a direct replacement for wheat flour in baked goods. Dietary changes may have an impact on gut microbiota (Korpela, 2018). Few studies examine the impact of substituting refined carbohydrate with wholegrain (Jebb, 2015). Overall, research suggests that a temporary dietary change is more likely to be successful and that this is unlikely to lead to a long-term dietary modification (Jebb, 2018). Similarly, it is unclear if temporary food waste reduction initiatives would translate into longer term actions.

Advance knowledge of consumer behavior would benefit the actors in the value chain. The potential benefits that foreknowledge of consumer behavior can have for the value chain include: prebreeders and breeders of grains receiving information about dietary choices so that nutritional characteristics of grains could be improved, farmers having information to support planting choices, grain processors and millers having time to reallocate resources to process other products preventing overproduction and other forms of value loss (Trollman & Trollman, 2019), food manufacturers being able to consider reformulation of their products to support both consumer health and nutrition as well as reflecting changes on the supply side of raw ingredients, and increased customer loyalty for the food retailer.

The main driver for selecting crowdsourcing platforms was independence from existing methods of tracking consumer behavior. Food retailers have loyalty apps and cards, but hesitate to share this information for various reasons including consumer trust and privacy, competitive advantage, and legislation. Edge computing would localize individual consumer data for greater security and enable faster processing by supporting AI. Food retailers do not all serve the same markets in terms of characteristics such as wealth, health, and motivations. Consumer trust may also be increased if the crowdsourcing is local and coordinated by a retailer they know.

The effectiveness of the food choice derivatives will depend on how they are perceived by the market. Although the design of the food choice derivatives ensures that they satisfy market regulations and have underlying physical resources, when the time comes for consumers to limit their consumption or make alternative choices, they may not comply. The degree of non-compliance may over time (through learning) be anticipated by AI. It will likely fall to retailers to determine if and what kind of sanctions may be imposed for non-compliance. Due to the localized nature of the nodes of the MAS, retailers may be able to share information that tracks whether consumers have made additional purchases elsewhere to make up for any commitments to reduce consumption. The independence and voluntary nature of participation may support data sharing as existing sales data are commercially sensitive and unlikely to be shared (Jebb, 2018).

This research may be applied to the commodity sector in general, including energy and metals. An example application is the energy reduction scheme in the UK intended to prevent electricity blackouts by allowing smart meter customers to reduce their usage during times of peak demand (Octopus Energy, 2022).

Future research directions should seek to explore and better understand how humanitarian responses in terms of reduced food consumption and waste impact on longer-term behavior and the most effective strategies for their realization.

## Conclusions

The purpose of this research was to design an alternative consumer-based price-making mechanism for derivatives markets to mitigate the negative effects of food speculation while improving food security and the sustainability of agricultural production. The design is presented in Fig. 4 with viability of the design supported by the case study of Ukrainian wheat based on the causal approach shown in Fig. 3. Although this “food choice” derivative is feasible, consumers should be supported in making relevant choices so that their nutrition and health is not compromised. Current communication within supply chains is not capable of managing the proposed derivatives which should instead make use of crowdsourcing platforms linked to futures markets with relevant data available to all actors of the supply chain to enable adequate time for planning. Enabling food choice derivatives has the potential to contribute to greater food security and decreased food waste when food systems are confronted with shocks.

## Data Availability

Not applicable.
